# Economic Analysis Aids Alcohol Research

**Published:** 2000

**Keywords:** economic aspects of AOD (alcohol or other drug) and AOD use, AOD price, sales and excise tax, cost of AODU (AOD use, abuse, and dependence) to business, social and economic costs and benefits of AOD, insurance cost due to AODU, cost-effectiveness of AOD health services, econometrics

## Abstract

Economic research contributes to our understanding of alcohol use and the prevention and treatment of alcohol-related problems in several ways. This article reviews three areas in which the tools of economic analysis have produced significant insights in recent years. First, economic researchers have analyzed the effects of beverage prices and taxation on alcohol consumption and on adverse consequences associated with alcohol use. Second, analyses of the costs and cost-effectiveness of treatment for alcohol use disorders have provided insight into the long-term costs and benefits of alternative approaches to alcoholism treatment. Finally, studies have incorporated economic techniques in estimating the overall magnitude of the burden placed on society by the misuse of alcoholic beverages.

The economic model of consumer behavior suggests that like other consumer goods, the demand for alcoholic beverages falls when prices rise. A large body of research shows that this “law of demand” holds for alcoholic beverages. This means that excise taxes and other public policies that affect the price of alcohol can influence the demand for alcohol. Because excessive alcohol consumption has adverse consequences for health and safety, studies of the consumer response to changes in alcoholic beverage prices are important.

## Effects of Changes in Alcohol Prices and Taxes

This section reviews recent economic research on the relationship between alcohol prices or taxes and alcohol consumption and related problems. For reviews of earlier research on these topics see Chaloupka 1993; Chaloupka et al. 1998; Cook and Moore 1993; Kenkel and Manning 1996; and Leung and Phelps 1993.

### Public Policies and Alcohol Prices

Public policies can affect alcoholic beverage prices in several ways. One way is excise taxes on alcoholic beverages. An excise tax is based on the quantity of alcoholic beverage purchased, in contrast to a sales tax, which is based on the price of a purchased good. The extent to which increases in excise taxes are passed along to consumers rather than absorbed by firms also determines the price of goods. Because little research has been conducted in this area, it is unclear how excise taxes influence prices for alcoholic beverages.

Some States exercise direct influence over alcoholic beverage prices by maintaining monopoly control over the sale of such beverages. Limited evidence suggests that alcoholic beverage prices have, on average, been about the same or only slightly higher in States with monopoly control (Nelson 1990) and that privatization has sometimes, but not always, resulted in lower prices (MacDonald 1986).

**Figure 1:**
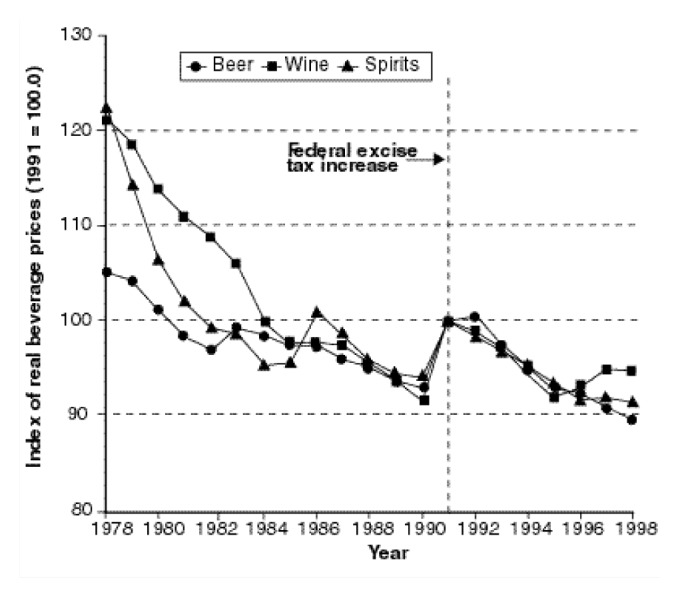
Inflation adjusted alcoholic beverage prices 1978–1996. SOURCE: Data obtained from the Bureau of Labor Statistics Web site (http://stats.bls.gov/sahome.html) December 1999.

When evaluating alcohol price and tax policies, it is important to consider the context provided by other public policies, private market forces, and general economic conditions. For example, alcohol excise tax rates are not routinely increased to compensate for the effects of inflation. As a result, the “real” (i.e., inflation-adjusted) tax rates have declined over most of the postwar period, except for the significant tax increase that took effect in 1991. This erosion of real tax rates has contributed to overall declines in real beverage prices over time (see figure 1).

### Alcohol Prices, Taxes, and Consumption

Although consensus exists among researchers that higher alcoholic beverage prices and taxes result in less drinking and fewer drinking-related problems, the magnitude of consumer response to price or tax changes is more difficult to determine. Economists measure consumer response to price changes by computing the *price elasticity*, defined as the percentage change in demand that results from a 1-percent change in price (see textbox, page 64). Price changes seem to affect the demand for beer less than they affect the demand for other alcoholic beverages. In 1993 researchers reported that a 1-percent increase in price translated into decreases in demand of 0.3 percent for beer, 1 percent for wine, and 1.5 percent for spirits (Leung and Phelps 1993).

**Measuring Consumer Response to Price Changes**

When the prices of goods rise or fall, the quantity of goods that consumers choose to purchase tends to change in response. Economists estimate the “price elasticity of demand” to measure consumers’ responsiveness to changes in prices. Estimates are computed with the following formula:

$$\text{Price elasticity}=\frac{\%\;\text{change in quantity demanded }(+\;\text{or }-)}{\text{change in price }(+\;\text{or }-)}$$

Example: A 5% price drop leads to a 10% increase in quantity demanded: $\frac{+10\%}{-5\%}=-2$

Some features of elasticity measures include the following:

- Price elasticities are negative for almost all goods, because consumers tend to choose to purchase greater quantities of goods at lower prices and fewer at higher prices.
- Elasticities of less than −1.0 indicate that demand is relatively responsive to changes in price (also called “elastic”). This is illustrated in the example shown.
- Elasticities in the range between −1.0 and zero indicate that demand is relatively unresponsive (also called “inelastic”). For example, if a price drops 5 percent and the quantity demanded increases only 2 percent, the price elasticity is −0.4.

**Figure 2:**
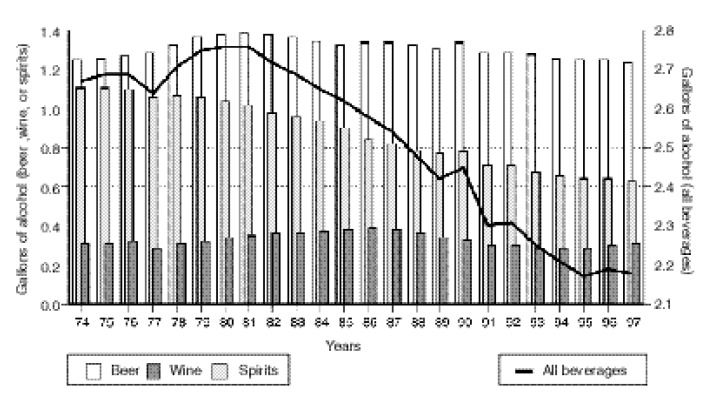
Per Capita alcohol consumption by beverage type, United States, 1974–1997. SOURCE: Nelson 1997. Reprinted with permission from *Empirical Economics*, Vol. 22, pp. 83–102, 1997 Copyright 1997, Springer-Verlag GmbH & Co., Heidelberg, Germany.

More recently, Nelson (1997) reported relatively unresponsive price elasticities of −0.16 for beer,−0.58 for wine, and −0.39 for spirits, with −0.52 for an overall price elasticity. His analysis also provided possible explanations for the decline in per capita consumption of alcohol (see figure 2) despite a decline in the real prices of alcoholic beverages in the United States in the same period (see figure 1). Nelson’s study showed that the demographic shift to an older population—which consumes less alcohol—outweighed the impact of falling real prices. Other factors, such as a shift to healthier lifestyles, also may help explain the decrease in consumption.

### Demand for Alcohol by Youth and Young Adults

A number of recent studies have used individual-level data to focus on alcohol demand by youths and young adults, who are considered at particularly high risk for alcohol problems. One study used survey data from the national Monitoring the Future (MTF) Study of high school seniors to explore the determinants of alcoholic beverage demand among young adults (Grossman et al. 1998). This study followed more than 7,000 people from 1976 to 1985 and tested an innovative theory of the demand for addictive goods (Becker and Murphy 1988). Previous research had accounted for habit formation by exploring past consumption of alcohol as a possible determinant—through acquired taste or addiction—of current consumption (see, for example, Andrikopoulos et al. 1997). The Becker and Murphy theory of addiction posits that consumers may anticipate that their current use of alcohol will influence their future demand for it. If so, expected future consumption is also a possible determinant of current alcohol demand, and factors that can be anticipated to affect future consumption also have an impact on current consumption choices. The policy implication of this theory is that the long-run demand for addictive goods is actually more responsive to price changes than the short-run demand (Grossman et al. 1998). The results suggest that raising alcohol prices would be an effective policy to reduce alcohol consumption among youth.

In contrast, another study found that beer taxes have a relatively small and statistically insignificant effect on teen drinking (Dee 1999). Using data from the MTF Study for 1977 through 1992, Dee examined the effects of minimum legal drinking age laws and beer taxes on the prevalence of teen drinking in three categories (1 or more drinks in the past month, 10 or more drinks in the past month, 5 or more drinks in a row in the past 2 weeks). The results suggested that raising the legal drinking age above 18 significantly reduced the number of high school seniors in each drinking category. However, within-State comparisons found beer tax rates to have no significant effect in reducing these drinking rates. Additional research is needed to clarify how taxes and other factors affect various patterns of drinking among different groups.

College students as a group are at particularly high risk for alcohol-related problems. To estimate alcohol demand for this population, Chaloupka and Wechsler (1996) merged drinking data from 17,000 college students with measures of beer prices and an index of drunk driving laws prevailing in the locations of the colleges. The results suggested that college students were less responsive to alcohol prices than other groups. The researchers did find, however, that more severe drunk driving penalties tended to reduce both drinking and binge drinking. These effects were found among underage and older students, both male and female.

## Alcohol Taxes and Traffic Fatalities

Research indicates that higher beverage taxes affect not only alcohol consumption but also various alcohol-related problems, such as traffic fatalities. Although the previous discussion suggests that overall demand for alcohol is only moderately responsive to price changes, a number of studies have found that higher alcohol taxes are linked to lower traffic fatality rates (Ruhm 1996; Phelps 1988; Kenkel 1993).

Ruhm (1996) found that for every 1 percent increase in the price of beer, the traffic fatality rate declined by nearly the same proportion. He found nearly identical results using fatalities per total vehicle miles driven. The study also showed that rates for nighttime fatalities and for people aged 18 through 20 were even more responsive to an increase in beer prices. This study, as well as a substantial body of prior research, suggests that a tax increase may be a useful tool to reduce traffic fatalities, particularly among youths and young adults. One recent study, however, has suggested that changes in fatality rates that have been attributed to beer taxes might be linked more strongly with other factors omitted from previous analyses (Dee 1999). Clearly, further research is needed.

In addition to investigating price effects among youths, researchers have studied price effects among other subgroups with a high risk of traffic accidents: binge drinkers and regular, heavy drinkers. Sloan and colleagues (1995) found that a 10 percent increase in the price of alcoholic beverages would decrease binge-drinking episodes (defined as consuming five or more drinks on one occasion in the past month) by eight percent. In addition, liability and insurance rules were more effective than criminal sanctions in reducing binge drinking. Another study found that persons who drank extremely heavily were unresponsive to price increases (Manning et al. 1995), suggesting that price increases would have a limited effect on traffic crashes among this group.

Overall, the evidence indicates that prices have modest effects on overall consumption and somewhat more substantial effects on traffic crash fatality rates. Small effects on consumption may have substantial effects on outcomes like traffic fatalities if, for example, higher prices reduce the number of drinks consumed on a given occasion of heavy drinking. Clarifying the nature of price effects on different aspects of consumption and on health-related outcomes remains a critical task for future research.

## Alcohol Demand and Marijuana Demand

The idea of using tax increases to reduce alcohol use raises concerns that such a policy may cause consumers to use less alcohol but increase marijuana use in response to increased beverage prices. Two recent studies have examined this issue, with contrasting results. One study found that alcohol and marijuana were economic complements (Pacula 1998), meaning that the goods tend to be used together, such as gin and tonic water. Thus, the researchers estimated that doubling the beer tax would reduce the use of marijuana as well as alcohol. This finding should be viewed with caution, however, because States with lower beer taxes may also have more tolerant social attitudes toward other substance use.

Another study found evidence that alcohol and marijuana were substitutes (i.e., an increase in the price of one causes a shift in consumption and an increase in demand for the other) (Chaloupka and Laixuthai 1997). The study found that raising both the price of beer and the minimum legal drinking age reduced youth demand for alcohol. Further, the results suggested that marijuana decriminalization reduced youth drinking. Under decriminalization, youth face lower potential costs of marijuana use, so the pattern found in this study suggested that youths substitute marijuana and use less alcohol in States where marijuana is decriminalized. In addition, the researchers found that higher marijuana prices increased alcohol demand, which is consistent with the conclusion that the two substances are substitutes.

Given the conflicting findings between these two studies, further research is needed to clarify the nature of the relationship between the demands for alcoholic beverages and marijuana.

## Benefits and Costs of Taxation

The bulk of research evidence shows that higher alcohol taxes or prices lead to reductions in alcohol consumption and in the adverse consequences of alcohol abuse. Studies of “optimal taxation” provide a framework for determining how heavily alcoholic beverages should be taxed by balancing the benefits of alcohol taxation with the costs that alcohol taxes impose on moderate drinkers and alcoholic beverage producers.

Several studies have concluded that substantial increases in alcohol taxes would yield social benefits (e.g., reductions in alcohol-related health problems) that exceed their costs (Manning et al. 1989, 1991; Pogue and Sgontz 1989). Other research (Heien 1995–1996), however, concluded that alcohol tax levels were too high. Heien suggested that this conclusion differed from those of previous studies for several reasons, including the timing of the study and its assumption that drinkers have lower health care costs than do nondrinkers.

However, assessing the net effects of alcohol consumption on health is difficult, and assessments may vary over the life span (Dufour 1996). For example, low-level alcohol consumption may generate net health benefits for some people, but even low levels of consumption may pose risks to others, such as teenagers (Dufour 1996). Further research is needed to explore the benefits and costs of alcohol taxation. For example, none of the studies mentioned in this section measured the potential benefits alcohol taxation may create by reducing violent behavior (Cook and Moore 1993).

Another important question is how the benefits and costs of alcohol taxation are distributed across the population. In assessing the fairness of a particular tax, one method is to consider the extent to which the burden of the tax falls disproportionately on lower income members of society. A tax that consumes a larger share of the income of poorer households is termed “regressive,” whereas a tax that consumes an increasing fraction of income as income rises is considered “progressive.” A study by the Congressional Budget Office (Sammartino 1990) found that, across households, expenditures on alcoholic beverages increased as income increased, but at a slower rate. As a result, lower income households paid less in alcohol excise taxes than did higher income households on average, but the taxes nevertheless consumed a larger proportion of income in lower income households. A more recent study (Lyon and Schwab 1995), found that alcohol taxes were still regressive, but slightly less so, when measured with respect to lifetime income instead of current income.

A related issue is employment and concerns that alcohol tax increases will hurt workers whose livelihoods depend on the production and sale of alcoholic beverages. However, the overall level of employment in the United States is determined by macroeconomic conditions, not adjustments in the tax rates on specific industries. A tax increase could cause a permanent job loss in the alcohol industry, but research on labor economics suggests that displaced workers would almost certainly find employment elsewhere eventually. Worker displacement remains costly not only during the period of unemployment but in the long term, because displaced workers appear to earn less on their new jobs (Jacobson et al. 1993; Ruhm 1991). These transitional costs should be included as an extra cost of increasing alcohol taxes, but most or all of the employment losses in the alcohol industry will eventually be offset by employment gains in other sectors of the economy (Kenkel and Manning 1996).

## Cost Research on Alcoholism Treatment

Relatively little research has been conducted on the cost of alcohol treatment, but important developments have occurred in recent years. Researchers are exploring whether people who undergo alcoholism treatment have lower health care expenditures afterwards and whether some treatment settings are more cost-effective than are others. Other questions are also being considered, such as whether shorter or longer periods of inpatient treatment are more cost effective and whether treatment cost savings in the short term might lead to a higher probability of relapse, and consequently, greater long-term treatment costs. Recent years have brought improvements in the methods used to analyze the costs of alcoholism treatment. These improvements hold considerable promise for the further development of the field.

## Research Findings

Early research on the cost of alcoholism treatment centered on general themes (see Jones and Vischi 1979; Annis 1986; Holder et al. 1991; and Finney et al. 1996 for reviews). Those themes included whether alcoholism treatment reduced overall health care costs, and whether such reductions were sufficient to cover treatment costs. More recent studies, discussed below, continue to examine other topics raised in earlier research. These include cost offsets, or the decrease in total health care costs after adjustment for alcohol treatment costs, and the cost-effectiveness of different treatments. The latest research focuses on new topics, such as the length of treatment and long-term costs.

### Cost-Effectiveness of Different Treatment Modalities

Finney and Monahan (1996), reanalyzed the cost-effectiveness literature originally evaluated by Holder and colleagues (1991). The newer study added 3 treatment modalities, bringing the total to 36, and used a procedure for assessing outcomes that rated the strength of each study’s findings on the basis of the research methods used. The reanalysis confirmed some of the findings of the original review, such as the effectiveness of some treatment modalities (e.g., social skills training, the community reinforcement approach, behavioral marital therapy, and stress management training) and the ineffectiveness of other modalities (e.g., residential milieu treatment and general counseling). Several treatment modalities, including brief motivational counseling, self-control training, and use of Antabuse (a drug that creates an aversive reaction to alcohol), were found less effective using the revised methods.

Overall, the range of effectiveness across all 36 modalities was reduced in the newer review. The reanalysis did not show a relationship between effectiveness and cost. When only those 26 modalities that had been documented by three or more studies were included, greater cost was related to lower effectiveness, but this relationship was not statistically significant.

Later research examined the costs of specific treatment modalities. In one study, investigators calculated the costs for each of the three treatments compared in a project called Matching Alcohol Treatments To Client Heterogeneity (Project MATCH) (Cisler et al. 1998). Project MATCH was an 8-year, multi-site clinical trial sponsored by NIAAA that investigated cognitive-behavioral therapy (CBT), motivational enhancement therapy (MET), and 12-step facilitation (Project MATCH Research Group 1997). Each of the therapies produced generally comparable treatment outcomes, raising the question whether any of these equally effective treatments could be offered for a lower cost. Findings showed that average per-patient costs for MET were the lowest, at $537, compared with $904 for CBT and $956 for 12-step facilitation. The number of patient contact hours differed across the therapies, from 4 hours for MET to 12 hours for both 12-step facilitation and CBT. When costs were computed per hour of patient contact rather than per patient, MET was actually more expensive ($134 per contact hour) than either CBT ($75 per contact hour) or 12-step facilitation ($80 per contact hour).

Another study compared treatment costs over a 3-year period for alcoholics who chose to attend Alcoholics Anonymous (AA) with those who sought professional outpatient treatment (Humphreys and Moos 1996). The study found that treatment costs were lower for the AA group over the course of the study and that outcomes were similar for both groups, indicating that voluntary AA participation may significantly reduce treatment costs without compromising outcomes.

### Cost Offsets

Recent studies also have continued to investigate cost offsets, or net reductions in health care costs attributable to alcoholism treatment. One study of health insurance claims generated by employees and dependents who received alcoholism treatment showed that after the initiation of treatment, health care costs incurred by alcoholics declined, but that differences in these costs from pre-treatment levels were relatively modest (Goodman et al. 1997). The researchers found that cost offsets were greater for clients who initially received inpatient rather than outpatient treatment.

Another research group examined the effect on legal costs, along with health care costs, of behavioral marital therapy for alcoholism treatment patients (O’Farrell et al. 1996*a*,*b*). The results are only suggestive, because of the small number of subjects included in the study. The analysis indicated that behavioral marital therapy decreased both health care and legal costs and that the savings exceeded the cost of delivering the therapy. Behavioral marital therapy was not found to be more cost-effective in prolonging abstinence from drinking than was simple individual counseling, but was just as cost-effective as individual counseling in promoting marital adjustment. In addition, when special sessions to prevent relapse were added to behavioral marital therapy, improvements occurred in abstinence from drinking and marital adjustment outcomes. The additional relapse prevention therapy did not, however, lead to greater savings in health care or legal costs (O’Farrell et al. 1996*b*).

### Length of Treatment

Although the relative merits of inpatient versus outpatient treatment continue to be examined (Long et al. 1998), most observers seem to have accepted the conclusions of Finney and colleagues (1996) that outpatient treatment should be encouraged for most patients, but access to inpatient treatment should be retained for those patients who need it. The focus of cost-effectiveness research has accordingly shifted from the issue of inpatient versus outpatient care toward consideration of other treatment program dimensions, such as shorter versus longer periods of treatment.

One research group used information from 98 U.S. Department of Veterans Affairs (VA) inpatient treatment programs to identify the characteristics of the most cost-effective clinics (Barnett and Swindle 1997). Their principal outcome measure was whether patients were readmitted to treatment at any VA hospital in the United States within 180 days of discharge. They found that both treatment cost and outcome were related to program size, intended length of stay, ratio of staff to patients, and client treatment histories. In addition, they concluded that 21-day programs were more cost-effective than 28-day programs.

A 1998 study of 12 inpatient alcoholism treatment facilities for U.S. Navy personnel yielded similar results (Trent 1998). A planned reduction from a 6-week to a 4-week treatment program allowed researchers to conduct a natural experiment of treatment outcomes under the two plans. Patients treated in the 4-week program achieved outcomes similar to those treated in the 6-week program. The researchers also noted that participation in aftercare (principally attendance at AA) was the best predictor of treatment outcomes at 1-year follow-up.

### Long-Term Costs

Alcoholism is a chronic disease. It is therefore reasonable to expect that any person with alcoholism may experience several episodes of treatment, separated by periods of sobriety, over the course of a lifetime. Therefore, treatment cost research examines the long-term, or lifetime, costs for affected individuals. Such research may determine if saving money in the near term is shortsighted because such savings lead to greater costs over the long run. For example, although inpatient treatment may not seem cost-effective in the short term, if it reduces episodes of later care, it may compare favorably with other treatment strategies over the long term.

Cost researchers are starting to investigate long-term costs. One research group has distinguished between the alcohol treatment costs incurred during the first 6 months of treatment and costs incurred later (Goodman et al. 1996). One such study of 879 insured employees and retirees who underwent alcoholism treatment found that the treatment setting (inpatient vs. outpatient) during the first 6 months had no bearing on either the need for or the total costs of later treatment (Goodman et al. 1996). Moreover, the intensity of treatment during the first 6 months had no effect on later treatment costs for patients diagnosed as alcohol abusers, although more intense treatments in the initial 6 months slightly reduced later treatment costs among patients diagnosed as alcohol dependent. Treatment after the 6-month mark was more common among alcohol-dependent patients (as opposed to alcohol abusers) and those who also abused other drugs. Treatment costs beyond the first 6 months were greater for those with drug abuse problems, liver disease, or coexisting psychiatric disorders, largely because these factors increased the likelihood that long-term treatment would occur in an inpatient rather than an outpatient setting.

These results seem to indicate that near-term savings can be achieved without triggering greater costs in the long run. This finding runs counter to an earlier finding that returning to treatment (over a 2-year window) was less likely among patients initially treated in an inpatient hospital setting than among those attending AA (Walsh et al. 1991). The tradeoff between near-term and later treatment costs clearly requires continued research attention.

## New Developments in Measuring Costs

Perhaps the most important new direction of recent studies is the development of improved methodological tools for conducting cost research. Previously, treatment cost studies have generally not been based on recognized economic principles for assessing cost. In addition, comparison of results across studies has been difficult. Improving research methods and increasing standardization will help advance this area of research.

Three significant recent developments in the improvement of cost measurement methodologies have been (1) the guidelines contained in the U.S. Public Health Service’s (PHS) Cost-Effectiveness in Health and Medicine (Gold et al. 1996; see also Russell et al. 1996; Siegel et al. 1996; Weinstein et al. 1996); (2) the Drug Abuse Treatment Cost Analysis Program (DATCAP) developed by French and colleagues (French and McGeary 1997; French et al. 1997); and (3) the Uniform Accounting System and Cost Reporting for Substance Abuse Treatment Providers, a contract product developed for the Center for Substance Abuse Treatment by Capital Consulting Corporation (Caliber Associates 1998*a*,*b*).

The PHS guidelines contain a set of recommendations for conducting cost-effectiveness studies (Gold et al. 1996; see also Russell et al. 1996; Siegel et al. 1996; Weinstein et al. 1996). Included in these guidelines are recommendations to measure costs to the entire society rather than from the perspective of a given treatment-delivering organization; to include a “reference case” in research reports or an analysis conducted according to a common, standard set of economic assumptions to facilitate comparison with other studies; and to identify ethical problems that may arise in the course of analysis.

The DATCAP takes a different approach (French and McGeary 1997; French et al. 1997). Its intent is to provide a procedure for measuring substance abuse treatment costs without placing a substantial burden on the treatment center staff. The procedure measures the market value of all goods and services expended in providing treatment. Costs are estimated from the perspective of the provider organization rather than from the perspective of the client, of third-party payers (such as insurance companies), or of the society at large. These cost-estimating procedures have been applied to employee assistance programs (Bray et al. 1996; French et al. in press) and to drug abuse treatment programs (French et al. 1996, 1997), but applications specific to alcoholism treatment have not yet appeared in the literature.

The Uniform Accounting System and Cost Reporting for Substance Abuse Treatment Providers was also developed more as a tool for treatment providers than for academic researchers (Caliber 1998*a*,*b*). Like DATCAP, it measures costs from the perspective of the provider organization. The Uniform System differs from DATCAP by focusing on accounting costs, which are based on a treatment program’s actual expenditures for goods and services used in providing treatment. These differ from economic costs (market value costs) whenever the treatment provider has access to free or subsidized resources, such as volunteer labor, the use of free or subsidized space, or donated food (Dunlap and French 1998). The purpose of a study and the perspective of its authors determine which of the two systems is more desirable. Most treatment providers would probably be more comfortable with accounting costs, as these most closely resemble the budgets that will be needed to provide the services. Researchers, on the other hand, are more likely to prefer economic costs, since conclusions based on the comparison of costs between programs should not be confounded by uneven access to free or subsidized resources.

By providing templates for the measurement of treatment costs, the above three systems promise to facilitate future research by (1) making any cost study easier to conduct by providing model cost-measurement systems and (2) providing standardization that should enable and encourage comparison between studies.

## The Economic Costs of Alcohol Abuse

The burden imposed by a disease can be measured in many ways, including the number of deaths attributed to it, the total number of cases, the number of new cases that occur in a given year, hospitalization rates, potential years of life lost, and other measures that combine mortality and quality-of-life information. Another approach to assessing the burden of disease is to estimate the associated “cost of illness” (or COI), which expresses the multidimensional impact of a health problem in dollars. A COI study of a particular health problem usually includes estimates of the costs of health care services, losses in productivity from illness and premature death, and other expenditures and resource losses that can be attributed to the health condition. Estimates for different diseases often are not directly comparable to one another because of variations in methods, data sources, and underlying assumptions (National Institutes of Health 1997).

Over the past two decades, five major studies have used the COI framework to estimate the economic costs of alcohol abuse^1^ in the United States (Berry et al. 1977; Cruze et al. 1981; Harwood et al. 1984, 1998; Rice et al. 1990). These studies estimate the costs of alcohol abuse including health care costs, productivity losses, and additional costs, such as those associated with alcohol-related crime and motor vehicle crashes. In the most recent of these COI studies, researchers estimated the overall economic cost of alcohol abuse at $148 billion for 1992, the most recent year for which adequate data were available at the time of the study (Harwood et al. 1998). Making adjustments for population growth and inflation, the authors also projected their estimates forward to 1995, for which the overall estimated cost was $166.6 billion, and to 1998, for which the overall estimated cost was $184.6 billion, or roughly $683 for every man, woman, and child living in the United States in 1998 (Harwood 2000). Unless otherwise noted, cost figures reported in this section are drawn from the update for 1998.

More than 70 percent of the estimated costs of alcohol abuse were attributed to lost productivity ($134.2 billion), most of which resulted from alcohol-related illness or premature death. The remaining estimated costs included health care expenditures to treat alcohol use disorders and the medical consequences of alcohol consumption ($26.3 billion, or 14.3 percent of the total); property and administrative costs of alcohol-related motor vehicle crashes ($15.7 billion, or 8.5 percent); and various criminal justice system costs of alcohol-related crime ($6.3 billion, or 3.4 percent). A breakout of the estimated costs for 1992 and the associated projections for 1998 are shown in the table.

Before the latest report, the economic costs of alcohol abuse were last estimated in 1990 using data for 1985 (Rice et al. 1990). The estimate by Harwood and colleagues for 1992 is 42 percent greater than the estimate by Rice and colleagues, even after accounting for expected increases due to inflation and population growth. However, the estimate for 1992 is almost exactly equal to the average of the estimates from four other major studies, the Rice study included, dating back to 1977 (adjusting each of the earlier estimates for inflation and population growth). Although the estimates for 1985 and 1992 were developed using similar approaches, Harwood estimated that more than 80 percent of the increase reported in the newer study could be attributed to differences in data and methodology rather than to real increases in alcohol abuse or its consequences.

## Distribution of the Burden of Costs

Harwood and colleagues (1998) included in their report an estimate of how the burden of the costs of alcohol abuse is distributed across various segments of society. This analysis, based on the data for 1992, estimated that about 45 percent of the estimated total cost was borne by alcohol abusers and their families, almost all of which was the result of lost or reduced earnings. About 20 percent of the total estimated cost of alcohol abuse was borne by the Federal government, mostly in the form of reduced tax revenues resulting from alcohol-related productivity losses, and 18 percent of the total was borne by State and local governments, in the form of reduced tax revenue and criminal justice and motor vehicle-related costs. Private insurance arrangements (including life, health, auto, fire, and other kinds of insurance) shouldered 10 percent of the total estimated cost, primarily in the areas of health care costs and motor vehicle crashes. Six percent of the total cost was borne by victims of alcohol-related crimes (including homicide) and by the nondrinking victims of alcohol-related motor vehicle crashes.

## Components of the Cost of Alcohol Abuse

The total estimated costs of alcohol abuse, constructed from estimates of numerous smaller categories, group into two main kinds of cost: the health care costs of alcohol abuse and productivity losses. Estimates for 1998 placed the health care costs of alcohol abuse at $26.3 billion (comprising 14.3 percent of the total estimated cost of alcohol abuse). These estimates include both the costs of treating alcohol abuse and dependence ($7.5 billion), and the costs of treating the various adverse medical consequences of alcohol consumption ($18.9 billion). Productivity losses, estimated at $134.2 billion (72.7 percent of the total) in 1998, includes losses from alcohol-related illness ($87.6 billion), premature death ($36.5 billion), and crime ($10.1 billion). Other factors contributing to the total estimated costs of alcohol abuse include insurance and legal costs ($15.7 billion) and the legal, property, and administrative costs of alcohol-related crime ($6.3 billion).

## Limitations and Caveats

As with earlier studies of economic costs, the latest research confirms that alcohol abuse imposes a heavy burden on society. Although researchers estimating the economic costs of alcohol abuse attempt to be as comprehensive as possible, and although the magnitude of costs revealed in these estimates is undeniably enormous, there are several important caveats that apply to the interpretation of these estimates.

First, the estimates should not be considered precise measures. Good data are not readily available for many of the areas in which costs are incurred. Some areas, such as productivity losses, employ quantities that are fundamentally unobservable, and thus must be based on theoretical and statistical inference. Second, these estimates are not able to capture all the significant aspects of the alcohol-related burden. Foremost among these is the human suffering endured by individuals with alcohol-related problems and their families. Finally, these estimates of the economic costs of alcohol abuse are not sufficient by themselves to justify the use of one method over another to reduce such costs. Other factors must be considered before any cost-reducing measures can be taken; the estimates are merely one piece of a larger puzzle.

## Conclusion

Additional alcohol research using economic analyses is needed to complement the strides that have already been made. For example, recent studies have addressed how alcohol prices and taxes influence alcohol consumption, confirming earlier findings that consumers respond to changes in the price of beer, wine, and spirits. Discrepancy still exists, however, as to how large those effects may be. The weight of evidence suggests that the effects are relatively modest, with a 1-percent increase in price expected to lead to less than a 1-percent decrease in consumption. Other studies have addressed whether higher alcohol prices or taxes reduce drunk driving and alcohol-related traffic fatalities. Recent research confirms that higher taxes can contribute to these public health goals. Improvements in methodology and data collection should enable future research efforts to reconcile the magnitudes of the estimated effects of taxes on consumption with the larger estimated effects of taxes on traffic fatalities. In addition, future research will need to clarify whether increases in alcohol prices or taxes can help reduce youth drinking, a population that is at special risk for alcohol-related problems.

Research has also shown that more expensive treatment does not necessarily lead to better outcomes. Also, research shows that cost offsets are achieved following treatment: health care cost reductions among those treated for alcoholism compensates sufficiently for the cost of the treatment. Researchers also have demonstrated that, while some patients will require inpatient therapy, for many patients, outpatient treatment may be more cost-effective. With such questions generally resolved, research is now focusing on other topics, such as comparing the cost-effectiveness of shorter versus longer inpatient treatment, and examining whether short-term savings from outpatient treatment are balanced against treatment costs that might be realized in the long term. Such research should benefit immensely from the increasing standardization of methods for measuring treatment costs.

Further research has confirmed that alcohol abuse imposes a heavy burden on society, with health care, treatment, productivity losses, premature death, crime, and legal costs in the billions of dollars. Researchers have estimated that 45 percent of these costs are borne by the abusers and their families, and 20 percent are borne by the Federal government. While researchers attempt to be comprehensive when estimating economic costs, estimates in areas such as productivity losses are based on statistical inference. Furthermore, the estimates cannot capture all the important aspects of the alcohol-related burden. As a result, other factors must be taken into account before any action is taken to reduce costs; estimates must be viewed as part of a larger whole.

## Figures and Tables

**Table:** Estimated Economic Costs of Alcohol Abuse in the United States, 1992 and 1998*

Economic Cost	1992 ($ millions)	1998 (Projected) ($ millions)
**Health care expenditures**		
Alcohol use disorders: treatment, prevention, and support	5,573	7,466
Medical consequences of alcohol consumption	13,247	18,872
		
**Total**	**18,820**	**26,338**
**Productivity impacts**		
Lost productivity due to alcohol-related illness	69,209	87,622
Lost future earnings due to premature deaths**	31,327	36,499
Lost productivity due to alcohol-related crime	6,461	10,085
		
**Total**	**106,997**	**134,206**
**Other impacts on society**		
Motor vehicle crashes	3,619	15,744
Crime	6,312	6,328
Fire destruction	1,590	1,537
Social welfare administration	683	484
		
Total	22,204	24,093
**Total costs**	**148,021**	**184,636**

* The authors estimated the economic costs of alcohol abuse for 1992 and projected those estimates forward to 1998, adjusting for inflation, population growth, and other factors.

** Present discounted value of future earnings calculated using a 6-percent discount rate.

SOURCES: Harwood 2000; Harwood et al. 1998.

## Footnotes

1 In this context, the term “alcohol abuse” refers to any cost-generating aspect of alcohol consumption. This differs from the clinical definition of the term, which involves specific diagnostic criteria. Thus, the costs associated with a single occasion of drunk driving that leads to injury or property damage would be counted in this framework, even though this behavior would not, by itself, meet the clinical criteria for a diagnosis of alcohol abuse.

## References

[b1-arcr-24-1-62] Andrikopoulos AA, Brox JA, Carvalho E (1997). The demand for domestic and imported alcoholic beverages in Ontario, Canada: A dynamic simultaneous equation approach. Applied Economics.

[b2-arcr-24-1-62] Annis HM, Stimmel B (1986). Is inpatient rehabilitation of the alcoholic cost-effective? Con position. Controversies in Alcoholism and Substance Abuse.

[b3-arcr-24-1-62] Barnett PG, Swindle RW (1997). Cost-effectiveness of inpatient substance abuse treatment. Health Services Research.

[b4-arcr-24-1-62] Becker GS, Murphy KM (1988). A theory of rational addiction. Journal of Political Economics.

[b5-arcr-24-1-62] Berry RE, Boland JP, Smart CN (1977). The Economic Cost of Alcohol Abuse–1975.

[b6-arcr-24-1-62] Bray JW, French MT, Bowland BJ, Dunlap LJ (1996). The cost of employee assistance programs (EAPs): Findings from seven case studies. Employee Assistance Quarterly.

[b7-arcr-24-1-62] Caliber Associates (1998a). Integrated Evaluation Methods: A Guide for Substance Abuse Treatment Knowledge Generating Activities.

[b8-arcr-24-1-62] Caliber Associates (1998b). Minimum Evaluation Data Set: Core Data Lists.

[b9-arcr-24-1-62] Chaloupka FJ (1993). Effects of price on alcohol-related problems. Alcohol Health & Research World.

[b10-arcr-24-1-62] Chaloupka FJ, Laixuthai A (1997). Do youths substitute alcohol and marijuana? Some econometric evidence. Eastern Economic Journal.

[b11-arcr-24-1-62] Chaloupka FJ, Wechsler H (1996). Binge drinking in college: The impact of price, availability, and alcohol control policies. Contemporary Economic Policy.

[b12-arcr-24-1-62] Chaloupka FJ, Grossman M, Saffer H (1998). the effects of price on the consequences of alcohol abuse. Recent Developments in Alcoholism.

[b13-arcr-24-1-62] Cisler R, Holder HD, Longabaugh R, Stout RL, Zweben A (1998). Actual and estimated replication costs for alcohol treatment modalities: Case study from Project MATCH. Journal of Studies on Alcohol.

[b14-arcr-24-1-62] Cook PJ, Moore M, Martin SE (1993). Economic perspectives on reducing alcohol-related violence. Alcohol and Interpersonal Violence: Fostering Multidisciplinary Perspectives.

[b15-arcr-24-1-62] Cruze AM, Harwood HJ, Kristiansen PL, Collins JJ, Jones DC (1981). Economic Costs to Society of Alcohol and Drug Abuse and Mental Illness, 1977.

[b16-arcr-24-1-62] Dee TS (1999). State alcohol policies, teen drinking and traffic fatalities. Journal of Public Economics.

[b17-arcr-24-1-62] Dufour MC (1996). Risks and benefits of alcohol use over the life span. Alcohol Health & Research World.

[b18-arcr-24-1-62] Dunlap LJ, French MA (1998). A comparison of two methods for estimating the costs of drug abuse treatment. Journal of Maintenance Addiction.

[b19-arcr-24-1-62] Finney JW, Monahan SC (1996). The cost-effectiveness of treatment for alcoholism: A second approximation. Journal of Studies on Alcohol.

[b20-arcr-24-1-62] Finney JW, Hahn AC, Moos RH (1996). The effectiveness of inpatient and outpatient treatment for alcohol abuse: The need to focus on mediators and moderators of setting effects. Addiction.

[b21-arcr-24-1-62] French MT, McGeary KA (1997). Estimating the economic cost of substance abuse treatment. Health Economics.

[b22-arcr-24-1-62] French MT, Dunlap LJ, Galinis DN, Rachal JV, Zarkin GA (1996). Health care reforms and managed care for substance abuse services: Findings from 11 case studies. Journal of Public Health Policy.

[b23-arcr-24-1-62] French MT, Dunlap LJ, Zarkin GA, McGeary KA, McLellan AT (1997). A structured instrument for estimating the economic cost of drug abuse treatment: The Drug Abuse Treatment Cost Analysis Program (DATCAP). Journal of Substance Abuse Treatment.

[b24-arcr-24-1-62] French MT, Dunlap LJ, Zarkin GA, Karuntzos GT The costs of an enhanced employee assistance program (EAP) intervention. Evaluation Program Planning.

[b25-arcr-24-1-62] Gold MR, Russell LB, Siegel J, Weinstein MC (1996). Cost-Effectiveness in Health and Medicine.

[b26-arcr-24-1-62] Goodman AC, Nishiura E, Hankin JR, Holder HD, Tilford JM (1996). Long-term alcoholism treatment costs. Medical Care Research Review.

[b27-arcr-24-1-62] Goodman AC, Nishiura E, Humphreys RS (1997). Cost and usage impacts of treatment initiation: A comparison of alcoholism and drug abuse treatments. Alcoholism: Clinical and Experimental Research.

[b28-arcr-24-1-62] Grossman M, Chaloupka FJ, Sirtalan I (1998). An empirical analysis of alcohol addiction: Results from monitoring the future panels. Economic Inquiry.

[b29-arcr-24-1-62] Harwood H (2000). Updating Estimates of the Economic Costs of Alcohol Abuse in the United States: Estimates, Update Methods and Data.

[b30-arcr-24-1-62] Harwood HJ, Napolitano DM, Kristiansen PL, Collins JJ (1984). Economic Costs to Society of Alcohol and Drug Abuse and Mental Illness: 1980.

[b31-arcr-24-1-62] Harwood H, Fountain D, Livermore G (1998). The Economic Costs of Alcohol and Drug Abuse in the United States, 1992.

[b32-arcr-24-1-62] Heien DM (1995–96). Are higher alcohol taxes justified?. CATO Journal.

[b33-arcr-24-1-62] Holder H, Longabaugh R, Miller WR, Rubonis AV (1991). The cost-effectiveness of treatment for alcoholism: A first approximation. Journal of Studies on Alcohol.

[b34-arcr-24-1-62] Humphreys K, Moos RH (1996). Reduced substance-abuse-related health care costs among voluntary participants in Alcoholics Anonymous. Psychiatric Services.

[b35-arcr-24-1-62] Jacobson LS, LaLonde RJ, Sullivan DG (1993). Earnings losses of displaced workers. American Economic Review.

[b36-arcr-24-1-62] Jones KR, Vischi TR (1979). Impact of alcohol, drug abuse and mental health treatment on medical care utilization: A review of the research literature. Medical Care.

[b37-arcr-24-1-62] Kenkel DS (1993). Drinking, driving, and deterrence: The effectiveness and social costs of alternative policies. Journal of Law and Economics.

[b38-arcr-24-1-62] Kenkel DS, Manning WG (1996). Perspectives on alcohol taxation. Alcohol Health & Research World.

[b39-arcr-24-1-62] Leung SF, Phelps CE, Hilton ME, Bloss G (1993). My kingdom for a drink...? A review of estimates of the price sensitivity of demand for alcoholic beverages. NIAAA Research Monograph No. 25.

[b40-arcr-24-1-62] Long CG, Williams M, Hollin CR (1998). Treating alcohol problems: A study of programme effectiveness and cost effectiveness according to length and delivery of treatment. Addiction.

[b41-arcr-24-1-62] Lyon AB, Schwab RM (1995). Consumption taxes in a life-cycle framework: Are sin taxes regressive?. Review of Economic Statistics.

[b42-arcr-24-1-62] MacDonald S (1986). The impact of increased availability of wine in grocery stores on consumption: Four case histories. British Journal of Addiction.

[b43-arcr-24-1-62] Manning WG, Emmet B, Keeler JP, Newhouse EM, Sloss EM, Wasserman J (1989). The taxes of sin: Do smokers and drinkers pay their way?. Journal of the American Medical Association.

[b44-arcr-24-1-62] Manning WG, Keeler EB, Newhouse JP, Sloss EM, Wasserman J (1991). The Costs of Poor Health Habits: A RAND Study.

[b45-arcr-24-1-62] Manning WG, Blumberg L, Moulton LH (1995). The demand for alcohol: The differential response to price. Journal of Health Economics.

[b46-arcr-24-1-62] National Institutes of Health (1997). Disease-Specific Estimates of Direct and Indirect Costs of Illness and NIH Support: 1997 Update.

[b47-arcr-24-1-62] Nelson JP (1990). State monopolies and alcoholic beverage consumption. Journal of Regulatory Economics.

[b48-arcr-24-1-62] Nelson JP (1997). Economic and demographic factors in U.S. alcohol demand: A growth-accounting analysis. Empirical Economics.

[b49-arcr-24-1-62] O’Farrell TJ, Choquette KA, Cutter HS, Floyd FJ, Bayog R, Brown ED, Lowe J, Chan A, Deneault P (1996a). Cost-benefit and cost-effectiveness analyses of behavioral marital therapy as an addition to outpatient alcoholism treatment. Journal of Substance Abuse Treatment.

[b50-arcr-24-1-62] O’Farrell TJ, Choquette KA, Cutter HS, Brown ED, Bayog R, McCourt W, Lowe J, Chan A, Deneault P (1996b). Cost-benefit and cost-effectiveness analyses of behavioral marital therapy with and without relapse prevention sessions for alcoholics and their spouses. Behavioral Therapy.

[b51-arcr-24-1-62] Pacula RL (1998). Does increasing the beer tax reduce marijuana consumption?. Journal of Health Economics.

[b52-arcr-24-1-62] Phelps CE (1988). Death and taxes: An opportunity for substitution. Journal of Health Economics.

[b53-arcr-24-1-62] Pogue TF, Sgontz LG (1989). Taxing to control social costs: The case of alcohol. American Economic Review.

[b54-arcr-24-1-62] Project MATCH Research Group (1997). Matching alcoholism treatments to client heterogeneity: Project MATCH posttreatment drinking outcomes. Journal of Studies on Alcohol.

[b55-arcr-24-1-62] Rice DP, Kelman S, Miller LS, Dunmeyer S (1990). The Economic Costs of Alcohol and Drug Abuse and Mental Illness: 1985.

[b56-arcr-24-1-62] Ruhm CJ (1991). Are workers permanently scarred by job displacements?. American Economic Review.

[b57-arcr-24-1-62] Ruhm CJ (1996). Alcohol policies and highway vehicle fatalities. Journal of Health Economics.

[b58-arcr-24-1-62] Russell LB, Gold MR, Siegel JE, Daniels N, Weinstein MC (1996). The role of cost-effectiveness analysis in health and medicine. Panel on Cost-Effectiveness in Health and Medicine. Journal of the American Medical Association.

[b59-arcr-24-1-62] Sammartino F (1990). Federal Taxation of Tobacco, Alcoholic Beverages, and Motor Fuels.

[b60-arcr-24-1-62] Siegel JE, Weinstein MC, Russell LB, Gold MR (1996). Recommendations for reporting cost-effectiveness analyses. Panel on Cost-Effectiveness in Health and Medicine. Journal of the American Medical Association.

[b61-arcr-24-1-62] Sloan FA, Reilly BA, Schenzler C (1995). Effects of tort liability and insurance on heavy drinking and drinking and driving. Journal of Law and Economics.

[b62-arcr-24-1-62] Trent LK (1998). Evaluation of a four- versus six-week length of stay in the Navy’s alcohol treatment program. Journal of Studies on Alcohol.

[b63-arcr-24-1-62] Walsh DC, Hingson RW, Merrigan DM, Levenson SM, Cupples A, Heeren T, Coffman GA, Becker CA, Barker TA, Hamilton SK, McGuire TG, Kelly CA (1991). A randomized trial of treatment options for alcohol-abusing workers. New England Journal of Medicine.

[b64-arcr-24-1-62] Weinstein MC, Siegel JE, Gold MR, Kalet MS, Russell LB (1996). Recommendations of the Panel on Cost-Effectiveness in Health and Medicine. Journal of the American Medical Association.

